# Expression of protein kinase A and the kappa opioid receptor in selected brain regions and conditioned place aversion in morphine-dependent rats

**DOI:** 10.18632/oncotarget.19671

**Published:** 2017-07-28

**Authors:** Xiuhua Song, Wenqiang Li, Yuzhong Shi, Jingdan Zhang, Yi Li

**Affiliations:** ^1^ Department of Psychiatry, Mental Health Center of Qingdao City, Qingdao, Shandong Province, China; ^2^ Department of Psychiatry, The Second Affiliated Hospital of Xinxiang Medical College, Xinxiang, Henan Province, China; ^3^ Department of Psychiatry, Affiliated Wuhan Mental Health Center, Tongji Medical College of Huazhong University of Science and Technology, wuhan, Hubei Province, China

**Keywords:** protein kinase A, kappa opioid receptor, conditioned place aversion, brain regions

## Abstract

This study examined adaptive changes in protein kinase A (PKA) and kappa opioid receptor (KOR) in selected addiction-related brain regions before and after conditioned place aversion (CPA). Seventy-two male SD rats were randomly assigned to an experimental group (morphine + naloxone, “MN”) and 2 control groups: MS (morphine + saline) and SN (saline + naloxone). MN rats were intraperitoneally injected with morphine twice per day for 6.5 days and naloxone once and trained to establish CPA model. MS and SN rats were injected with equivalent volumes of morphine plus saline and saline plus naloxone, respectively. PKA and KOR in AcbSH, CeA and VTA were measured by immunohistochemistry. Before CPA, there were no significant differences in PKA and KOR expression levels in the AcbSH, CeA and VTA between MN and 2 control groups. After CPA, significant differences in PKA expression were detected in the AcbSH (P<0.001) and VTA (P=0.018) between MN and 2 control groups. The average gray intensity of MN group (109.50±4.66) in AcbSH was significantly higher than that of MS (126.50±3.70, P<0.001) and MN (133.50±6.364, P<0.001) groups. Significant differences in KOR expression were also detected between MN and 2 control groups in CeA (P<0.001). In MN group, PKA and KOR expression levels showed adaptive changes at different points of CPA. These findings demonstrated that neuroadaptation mediated by PKA and KOR may be an important molecular neurobiology basis for CPA. The upregulation of AC-cAMP-PKA-CREB signaling pathway in AcbSH and VTA has some role in the neurobiological mechanism of CPA.

## INTRODUCTION

Opioid addiction is a chronic brain disease characterized by persistent and refractory compulsive drug-seeking behaviors. Its associated psychological and physical dependence often results in intolerable withdrawal symptoms, which further strengthen the impulsive drug-seeking behaviors and lead to relapse. Therefore, understanding the mechanisms underlying withdrawal symptoms is critical in the clinical management of opioid addicts.

The compulsive behavior of addiction is mediated by the reward craving pathway [1]. In drug abusers, there is a “drug abuse → addiction → withdrawal, which motivates disgust → relapse” cycle, leading to a vicious cycle of drug use [2, 3]. Thus, feelings of disgust, which are motivated by withdrawal symptoms, may be an important therapeutic target for preventing relapse.

AC-cAMP-PKA-CREB is one of the most frequently studied anatomical and neurochemical pathways for understanding withdrawal-induced aversion motives [4, 5]. Studies have shown that cannabinoids and opioids increase the activity of PKA via stimulating acetylate cyclase (AC) activities in the dopaminergic system. Elevated PKA was observed in the striatum and nucleus accumbens (NAC) in mice administered cannabinoids acutely, while PKA decreased in mice given a cannabinoid antagonist or dopamine receptor antagonists [6, 7], indicating possible adjustments under acute stress conditions following adaptation to environmental changes. Nevertheless, the impact of chronic administration of cannabinoid drugs on PKA activity remains unclear. One study showed that chronic administration of tetrahydrocannabinol (THC) to mice might increase PKA expression in the cerebral cortex [8]. However, findings on its impact on cerebellar PKA activity remain inconclusive.

In morphine-dependent rats, injection of Rp-cAMPS into the LC area and PAG reduced morphine withdrawal symptoms when naloxone was administered [9–12]. In our previous study, we also found that p-CREB AcbSH expression significantly increased in some brain regions including the VTA, CA1, LC, and PAG [13]. These findings imply that levels of certain mediators in the addiction pathway might be increased or reduced by opioid dependence, leading to a state of irritability and disgust and maintaining a negative addictive pathway.

Conditioned place aversion (CPA) is a widely used model in exploring the biological mechanisms underlying aversive motivation induced by acute and chronic opiate addiction withdrawal [14, 15]. In the formulation of the CPA model, after substance dependence withdrawal, feelings of disgust occur. Whether changes in the expression of PKA and KOR in certain brain regions are the molecular basis of CPA is unclear. To reveal the biological basis of morphine withdrawal aversion, this experiment established a CPA animal model and measured PKA and KOR protein expression in the core-shell area of the accumbens, amygdala central nucleus, and the ventral tegmental area (VTA) at different time points.

## RESULTS

### The development of CPA

**Figure 1:**
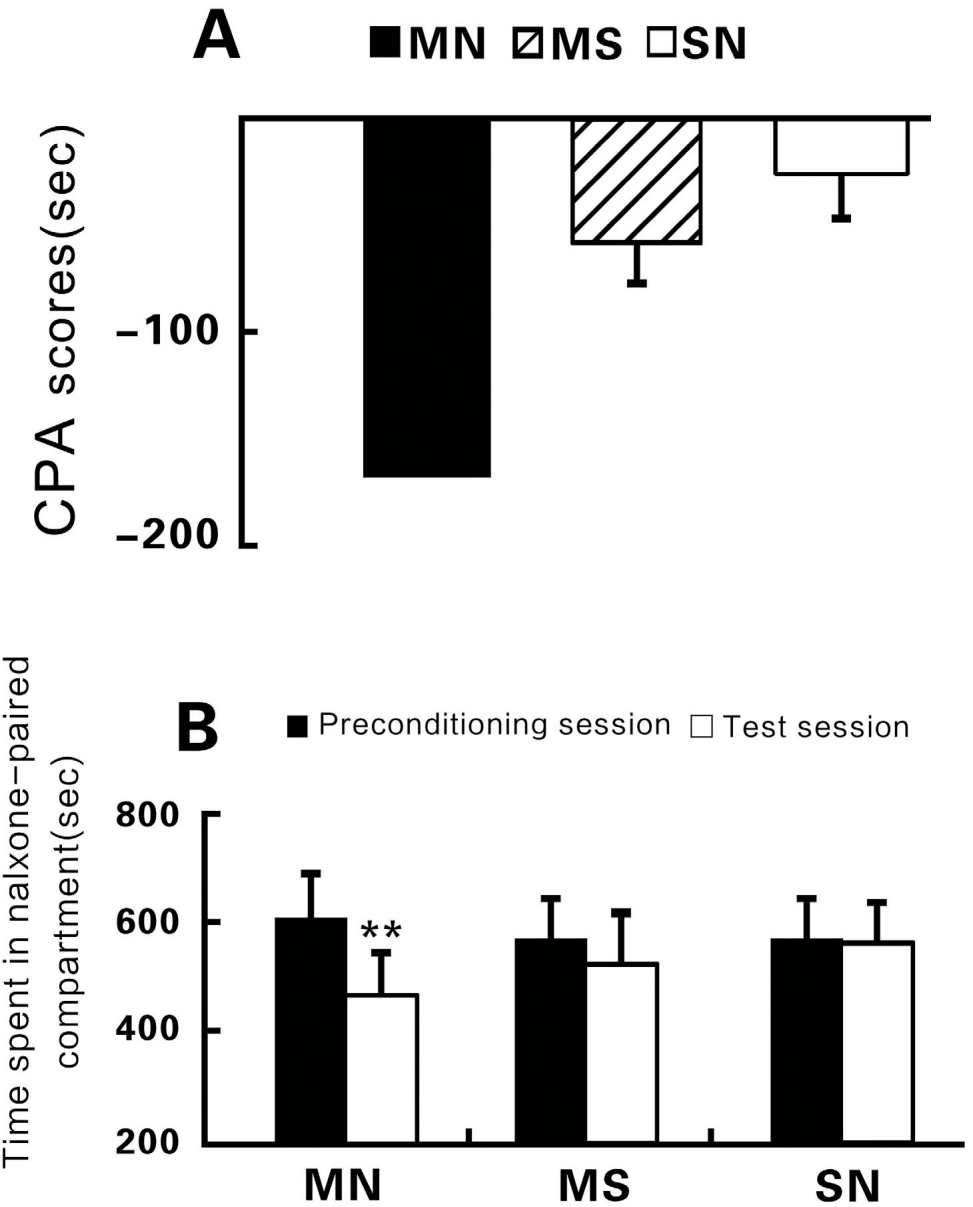
Changes in the time that animals stayed in the withdrawal paired compartment **(A)** Comparison of conditioned place aversion (CPA) scores. The CPA score is defined as the time in the withdrawal- paired compartment during the test session minus that during the preconditioning session. The numbers in parentheses indicate the numbers of animals in each group. **(B)** Comparison of the time animals stayed in the withdrawal-paired compartment between during the preconditioning session (closed bars) and test session (open bars). **P<0.01, independent-samples t-test.

After CPA, rats took much less time in the conditioning session in comparison with that in the preconditioning session (MN, n=24, test session: 445.67±42.40 seconds; preconditioning session: 608.60±50.70 seconds; t=9.008, P<0.01, test vs. preconditioning, Figure 1). The establishment of CPA depended on repeated morphine administration as MS animals also presented CPA to a certain degree (MS, n = 20, test session: 526.66±63.02 seconds, preconditioning session: 578.60±87.95 seconds; t= 1.503, P > 0.05, test vs. preconditioning, Figure 1), but SN animals had no any symptoms of CPA when comparing the time taken between the conditioned compartment during preconditioning and the test session (SN, n = 16, test session: 550.69±78.11 seconds; preconditioning session: 553.63±86.94 seconds; t=0.8, P>0.05, test vs. preconditioning, Figure 1).

### Somatic symptoms of naloxone-precipitated morphine withdrawal

Table 1 displays somatic signs of naloxone-precipitated morphine withdrawal. Somatic signs were more prevalent in the MN group than the MS and SN groups.

**Table 1:** Precipitated withdrawal signs on day 6

Withdrawal sign	Wet-dog shaking	Stretching	Rearing	Jumping	Diarrhea
MN group (n=24)	1.67±1.23^*^	1.22±0.67^*^	3.89±4.10	1.56±1.33^*^	22/24^*^
MS group (n=24)	0.67±0.71	0.00±0.00	1.89±1.83	0.00±0.00	0/24
SN group (n=24)	0.56±0.53	0.00±0.00	4.44±3.78	1.00±0.87	0/24
F	4.764	30.250	1.414	6.637	
P	0.017	0.000	0.263	0.005	

Note: Counted signs are shown as the mean ± standard deviation. Prevalence of diarrhea is expressed as the number of rats showing diarrhea to the total number of rats tested. Withdrawal sign is significantly different from 0 at level of significance 0.05.

### CPA extinction

**Figure 2:**
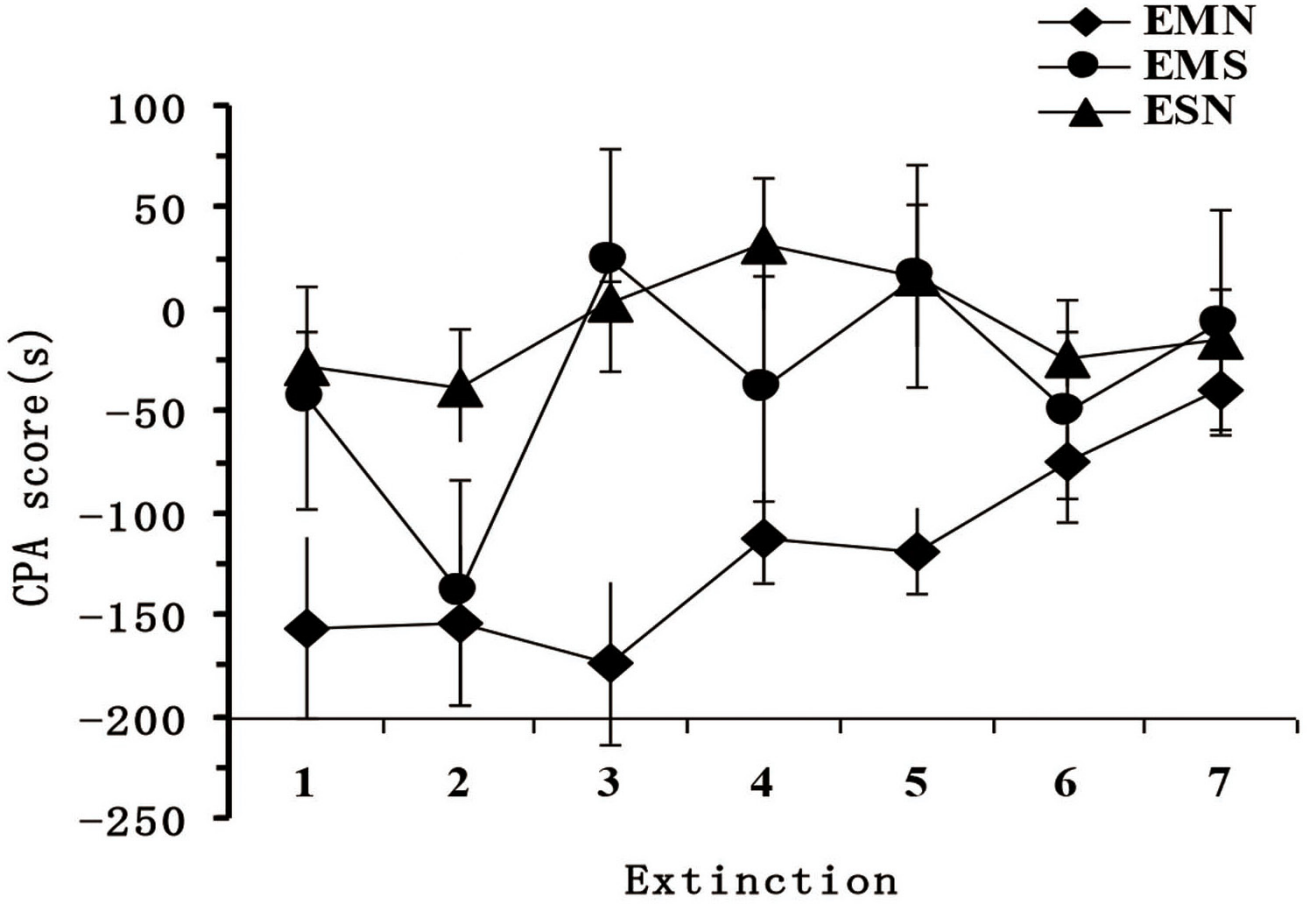
Changes in the CPA scores during the extinction phase

After training, the time associated with the drug-paired compartment in mice from the MN group gradually increased. CPA behavior subsided significantly (Figure 2).

### CPA reinstatement

**Figure 3:**
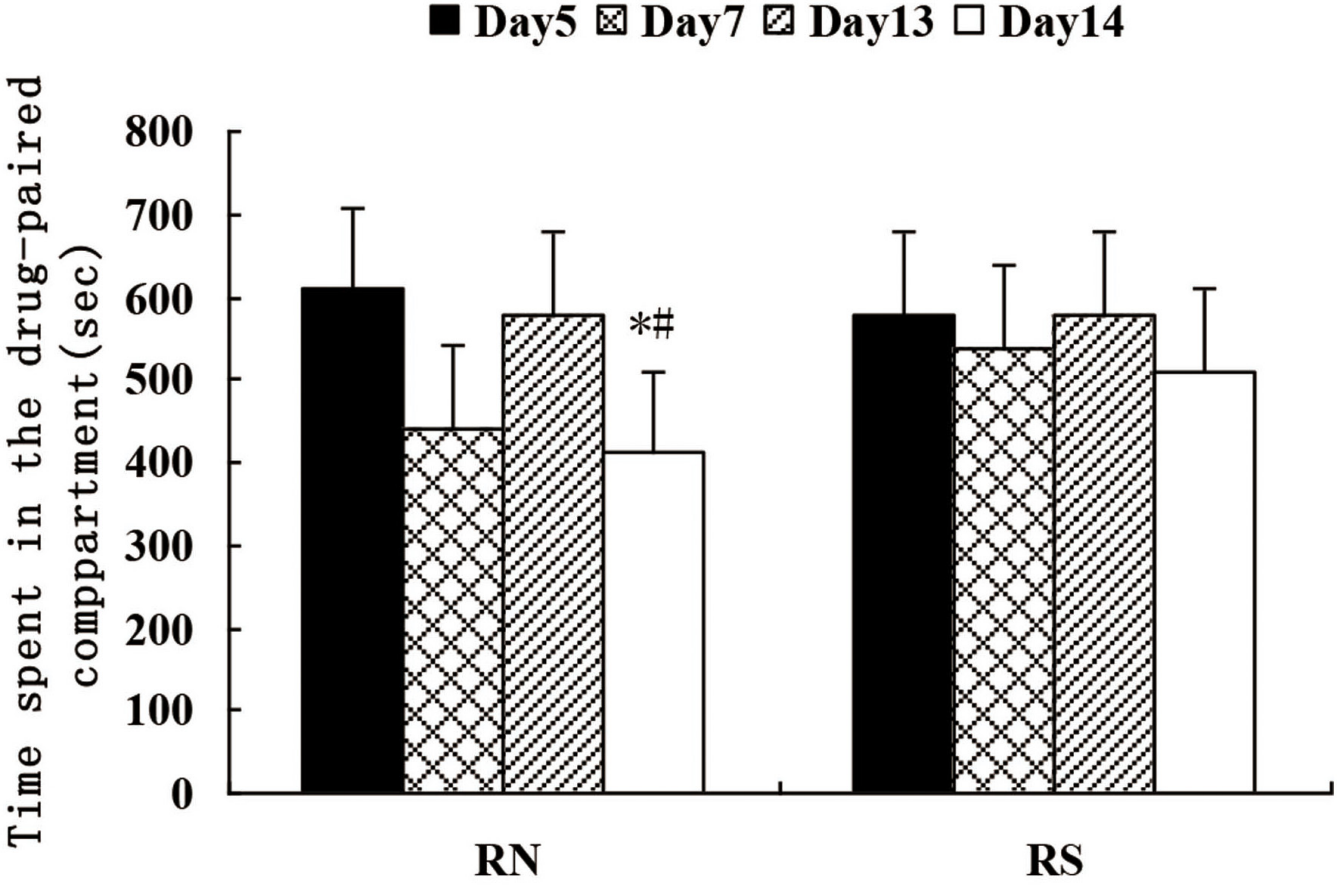
Comparison of the actual time rats stayed in the withdrawal-paired compartment on days 5, 7, 13 and 14

After drug kindling (morphine + naloxone), the time associated with the drug-paired compartment was reduced significantly. The CPA behavior again subsided significantly. The results of the time the mice spent in the drug-paired compartment did not show any significant difference between the MS and SN groups (Figure 3).

### Immunohistochemical results

**Figure 4:**
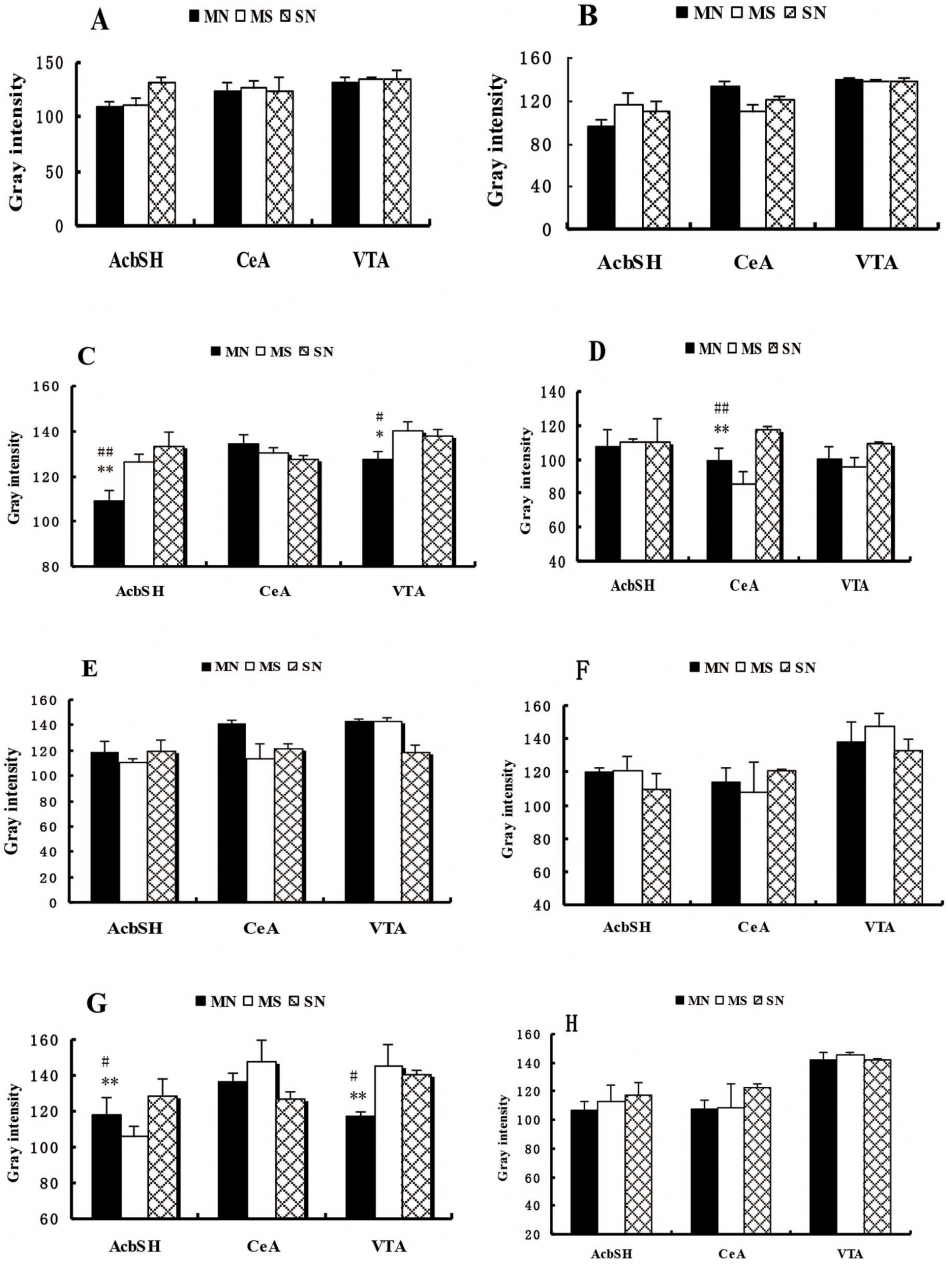
Each inspection brain regions PKA and kappa opioid receptor expression Each were seized before the establishment of the **(A)** CPA the brain regions PKA expression. **(B)** CPA before the establishment of various seized the brain regions Kappa opioid receptor expression. **(C)** CPA established various brain regions PKA expression was seized. **(D)** CPA established various brain regions Kappa opioid receptor expression was seized. **(E)** CPA subside after each examination of brain regions PKA expression. **(F)** CPA subsided after each seized the brain regions Kappa opioid receptor expression. **(G)** CPA reconstruction after each was seized the brain regions PKA expression. **(H)** CPA reconstruction after each seized the brain regions Kappa opioid receptor expression. Longitudinal axis, the gray-scale values (gray intensity) and positive expression was negatively correlated. Each group (n = 6), each sample slice randomly selected 4 immunohistochemical staining gray scale value (mean ± SEM), compared with the MS group: * P <0.05, ** P <0.01; compared with SN:# P <0.05, # # P <0.01 (ANOVA, LSD). (AcbSH: accumbens core-shell area, the CeA,: central amygdaloid nuclei, the VTA: ventral tegmental area).

After the establishment of CPA, PKA expression was much higher in the MN group than in control groups in the AcbSH and VTA, but no statistically significant difference was found in the CeA. KOR expression was much higher compared to the SN group in CeA, and no obvious changes were seen in the AcbSH and VTA. When CPA subsided, PKA and KOR expression showed no significant differences among the 3 groups in the AcbSH, CeA and VTA. When CPA reconstructed, PKA expression was significantly higher compared with the SN group in the AcbSH and VTA, but there was no significant difference in the CeA. KOR expression was not significantly different between the 3 groups in the AcbSH, CeA, and VTA (Figure 4).

**Figure 5:**
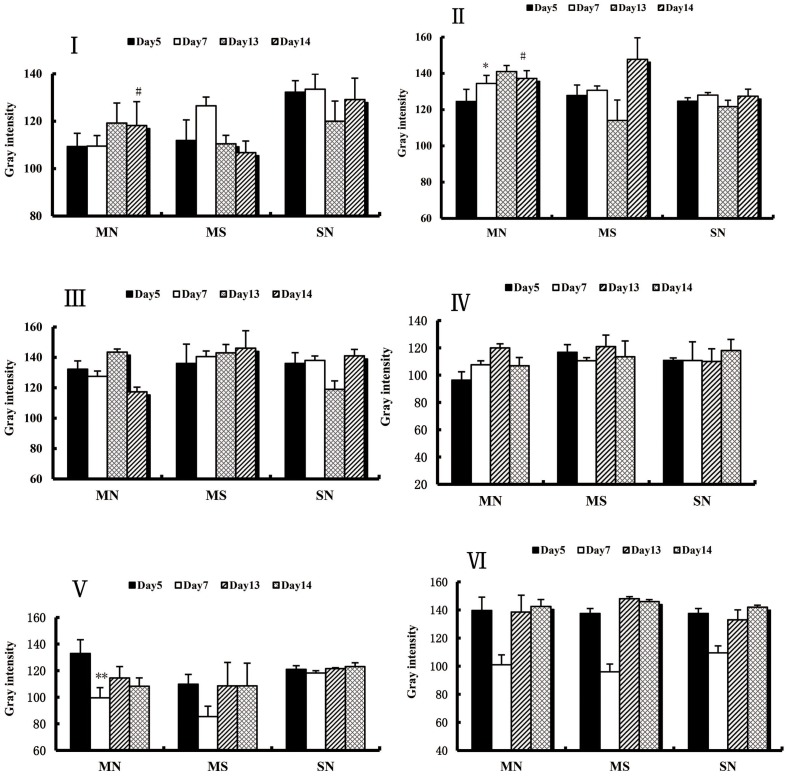
Various brain regions PKA and kappa opioid receptor expression was seized at each time point of the CPA (I) within the respective time points in the CPA AcbSH of PKA expression. (II) at each time point of the CPA the CeA of PKA expression. (III) in the CPA various time points VTA PKA expression. (IV) in the CPA time the point AcbSH within Kappa opioid receptor expression. (V) at each time point in the CPA the CeA within Kappa Opioid receptor expression. (VI) at each time point in the CPA of the VTA Kappa opioid receptor expression. Longitudinal axis, the gray-scale values (gray intensity) and positive expression was negatively correlated. Each group n = 6, each randomly selected the four immunohistochemical staining slices gray value (mean ± SEM). Day7 compared with Day5: * P <0.05, ** P <0.01; Day14 with Day5 compared:# P <0.05, # # P <0.01 (ANOVA, LSD). (MN groups: chronic morphine + naloxone reminder addiction group; MS Group: the chronic morphine + saline reminder addiction group; SN Group: chronic saline injection + naloxone reminder addiction group).

A direct comparison was also conducted between PKA and KOR expression at each time point of CPA in each brain region. In the MN Group, PKA expression in the AcbSH and CeA showed significant differences within each time point of CPA and did not change significantly in the VTA. KOR expression in the AcbSH, CeA and VTA showed significant longitudinal difference between each time point of CPA (Figure 5).

## DISCUSSION

This study showed that, in the CPA formation process, PKA expression changed adaptively to varying degrees in each brain region. After the establishment of CPA, PKA expression significantly increased in the AcbSH and VTA. In CPA established MN rats, KOR was highly expressed in the AcbSH, CeA and VTA.

### PKA expression during the process of CPA formation

This finding corresponded to adenylate cyclase 8 (AC 8) expression in a preliminary study. In the AC-cAMP-PKA-CREB pathway, adenylate cyclase (AC) catalyzes intracellular ATP of adenosine monophosphate (cAMP). PKA is not active as a tetramer. Adenylyl cyclase produces cyclic adenosine 3’, 5’-monophosphate (cAMP), which further activates PKA via binding to the regulatory subunits and causes the release and nuclear translocation of active catalytic subunits. The activity of PKA is related to elevated PKA catalytic subunit translocation. High expression levels of PKA protein may be one of the key factors established by CPA. Increased PKA activity can be observed during withdrawal from a variety of addictive drugs. Similarly, in rats withdrawn from cocaine, PKA activity was elevated in the NAc for the first 30 days, and returned to baseline level by 90 days after withdrawal [10]. In the chronic THC-treated mice, withdrawal precipitated by quick injection of a CB1 antagonist temporarily up-regulated PKA activity in the cerebellum and was related to elevated physical withdrawal symptoms [11]. In the present study, compared with the control group, the MN group showed more wet dog shakes, body stretching, jumping and diarrhea. PKA improved drug-induced synaptic plasticity through the phosphorylation of the GluR1 subunit of AMPA receptor, which was further transported to the cell membrane [16]. The dopamine D1 receptor agonist, SKF81297 or SCH23390, and the PKA activator, SpcAMPS, heightened the expression of GluR1 on cell surfaces in the rat AcbSH [17]. This result indicates that elevation of glutamate levels in the nucleus accumbens might be caused by increased synaptic glutamate release during withdrawal in morphine-dependent rats [18]. The above evidence shows that PKA might enhance neural plasticity of substance dependence withdrawal after CPA through this mechanism.

### KOR expression in different brain regions

It has been evident that the kappa opioid system plays a key role in cocaine dependence and that chronic cocaine use and withdrawal alter kappa opioid receptor (KOR) density [19]. Treated with the same procedures, rats of separated groups were withdrawn from cocaine or saline for 24 hours or 2 weeks. Nevertheless, in rats withdrawn for 24 hours from chronic cocaine, a rise in KOR-activated [35S] GTP binding in the VTA was observed. Findings from this study indicate a cocaine withdrawal-induced elevation of KOR signaling in the VTA, supporting the potentially important role of KOR in quick withdrawal from cocaine [19]. Recently, a pharmacological study found that application of KOR antagonists reduced cocaine self-administration [20]. The inhibition of KOR reduced the intake of cocaine in rats with extensive access to cocaine. Accordingly, KOR function in the limbic projection midbrain area changed after naloxone withdrawal, such as dynorphin/kappa opioid receptor systems exhibited inhibitory effects by inhibiting the release of DA in the reward pathway of the brain.

The KOR is a G protein-coupled receptor by which AC is activated into adenosine monophosphate (cAMP), which further activates PKA. The CREB undergoes phosphorylation by activated PKA, up-regulating physiological changes in genes such as the dynorphin gene. In the CPA model, MN group rats demonstrated obvious withdrawal signs, including wet dog shakes and body stretching, which might be related to anxiety-like behavior and aversive behaviors caused by the stress-regulating effects of KOR [21]. Many studies suggest that κ receptors may regulate learning and memory behavior [22]. The impact of stress on cognition is highly variable, mainly because deficits vary depending on the severity, type, and timing of stressors. However, there is evidence supporting the meditating role of stress-induced or exogenous activation of the KOR on cognition. For example, the rewarding valence of cocaine-associated cues could be induced by behavioral stress via a dynorphin/kappa opioid receptor-mediated mechanism [23]. Withdrawal made the rewarding valence extremely offensive. The motivation of getting rid of this negative sentiment prompted the rats craving for addictive substances and eventually resulted in relapse. As CPA subsided, this memory faded too. The aversion that was recorded in the mind would not be recalled until CPA was re-ignited, thus leading to a series of negative reinforcement processes.

After administration of naloxone to rats in the model group, craving behavior levels immediately reached a certain level where rats lost control over excessive drug intake, and this change was persistent. The biggest difference was the ability to adjust the drug intake as an outside temporary change compared with the control group. Fortunately, the brain of human adapted to pick up a normal plasticity and allowed learning ability to control substance use. On the other hand, the plasticity makes addicts involve in a downward spiral in drug-related stimulus, which no longer controls drug-seeking behavior, finally resulting in the uncontrollable substance use behavior of addiction [23, 24].

### Biological mechanisms underlying PKA changes

This elevated KOR activity resulted from substance use is in accordance with withdrawal-associated negative emotions in substance use. By antagonizing motivational withdrawal signs, using KOR antagonists may be helpful in managing drug dependence, thereby preventing or reducing addiction relapse. Therefore, once a state of drug addiction is developed, the inhibition of KOR may be effective in reducing the compulsive intake of drugs. As a result, the inhibition of the KOR system is a therapeutic target for blocking uncontrollable drug addiction and susceptibility to relapse of patients in or after recovery [25]. In addition, it has been demonstrated that activation of the KOR may exert an opposite impact on morphine-induced adverse symptoms. The blockade of KOR with selective antagonists may be a promising strategy to addiction relapse.

DA released post-synaptically connects G-protein-coupled receptors, which in turn activates the PKA pathway and further results in the phosphorylation of the CREB family of transcription factors [26]. Much evidence supports the key role of the pCREB in the strengthening learning [27]. In addition, there is an increase in pCREB in brain regions involved in stress-induced reinstatement (i.e., NAc and CeA), induced by acute and chronic stress [28]. In particular, CREB can be activated by cocaine within addiction-related brain regions (i.e., NAc, and VTA) [29]. Taken together, it is possible that that the disruption of CREB in the NAc affects motivation via reducing depressive-like states and facilitating reward [30].

The dynorphin/KOR system inhibits DA release of the reward pathway [31, 32]. This effect can be antagonized by KOR inhibitors [31]. In addition to DA, the AcbSH also receives glutamate from cerebral cortex projection fibers [33]. In keeping with previous findings, KOR activation increased phosphorylation of CREB in the AcbSH. In one recent study, we found that glutamatergic excitatory postsynaptic currents (EPSCs) can be inhibited by KOP receptor activation pre-synaptically in the NAc shell *in vitro*. Therefore, the KOR system may be potentially important in the development of novel treatments for dependence [34].

The reason that PKA and KOR expression after CPA was subsided and KOR expression after CPA was reconstructed did not show significant change might be related to the strains, size, category of the experimental animals and the experimental environment conditions or that the means of detection were not able to identify this kind of change. Another concern was that the PKA and KOR expression changes might occur at the mRNA level rather than at the protein level or both might be out of sync. In addition, there may be a change in the functional status of the two proteins.

In addition, the prodynorphin/KOR system is greatly plastic. In the NAc, levels of dynorphin mRNA and protein are increased after using drugs. Importantly, KOR levels may have been changed due to exposure to cocaine or amphetamine.

In summary, these findings demonstrated that neuroadaptation mediated by PKA and KOR may be important to the molecular neurobiological basis of CPA. The upregulation of the AC-cAMP-PKA-CREB signaling pathway in the AcbSH and VTA plays some role in the neurobiological mechanism of CPA. These changes may be important to the behavioral responses to drug addiction.

## MATERIALS AND METHODS

### Animal preparation

All experiments were performed with male Sprague Dawley rats (weight: 180–220 g per rat). These rats were habituated in their housing environment for 1 week before the experiment. Rats were group-housed, three or four per cage, with free access to water and food and given a 12-hour light/dark cycle (lights on from 08:00 hours until 20:00 hours) in a thermoregulated environment. All experiments were conducted according to the guidelines of the International Association for the Study of Pain, and every effort was made to minimize both the animal suffering and the number of animals used.

### Conditioning box

The conditioning box [35] (width: 30 cm; length: 60 cm; height: 30 cm) was divided into two equal-sized compartments with distinctive visual color and floor texture. One compartment was black with a smooth floor; the other compartment was white with a textured floor. The box was sound and light attenuated under conditions of dim illumination (40 lux) and masked to white noise. The time that rats spent in each compartment was automatically recorded using a video camera mounted to the ceiling of the box.

### Experimental procedure

#### Grouping and pretreatment

Rats were randomly divided into 3 groups: chronic morphine injection and naloxone-precipitated withdrawal (MN group, n = 24), chronic morphine injection and saline-precipitated withdrawal (MS group, n =24), and chronic saline injection and naloxone-precipitated withdrawal (SN group, n = 24); the latter 2 groups were control groups. For MN and MS groups, rats were administered morphine (First Pharmacy Manufacturer of Shenyang, No. 100305-1, Shenyang, China) intraperitoneally (i.p.), 10 mg/kg, twice a day at 08:00 and 20:00 for 6.5 days, from morning of day 1 to day 7. Rats in the SN group were given an equal volume of saline treatment using the same procedure [36, 37].

### Preconditioning session

The experimental process consisted of 3 distinct sessions: a preconditioning, conditioning, and test session. On day 4 of the morphine or saline treatment, the partition separating the two compartments was replaced with a divider that had a 10×10 cm gap, which allowed the rats to explore the box freely. The rats were individually placed at the boundary of the compartments and allowed to explore the box freely for 900 seconds as a habituation procedure. On day 5 (preconditioning session), the same trial was performed, and the time spent in each compartment during 900 seconds was recorded.

There was no significant difference between the time spent in the black compartment [468.19±60.55 seconds, n=60 (final numbers used in the behavioral test)] and the white compartment (431.81±60.55 seconds, n=60), indicating that there was no preference bias before conditioning. However, we used a bias-like protocol to nullify the initial preference of each rat by choosing the conditioning side as the side the rats spent more than half of the total time, i.e., >450 seconds, as previously discussed. Rats that spent > 80% of the time (> 720 seconds) in one side on day 5 or showed a time difference > 200 seconds between the two sides on day 4 and day 5 were excluded. In total, 11 rats were excluded.

### Conditioning session

On day 6, place conditioning was performed. At 10:00, 2 hours after the morphine or saline injection, each rat was injected with saline (1 ml/kg i.p.) and then confined to its non-preferred compartment for 30 minutes. After 4 hours, rats in the MN and SN groups were injected with naloxone (0.3 mg/kg i.p., Beijing Four-ring Pharmaceutical Co., No. 20100207, Beijing, China) and rats in the MS groups were injected with an equal volume of saline. The rats were then confined to their preferred compartment (naloxone-precipitated withdrawal-paired compartment) for 30 minutes.

### Test session

At 10:00 on day 7, the partition was removed, and rats were individually placed at the compartment boundary and allowed to explore the box freely. Time spent in each compartment during 900 seconds was recorded.

### Extinction procedure

During the test session on day 7, rats were placed in the box and allowed to explore the 2 compartments freely, without any injection. From day 8 onward, these rats received 2 extinction training sessions per day, one in the morning and the other in the afternoon. In each trial, rats were placed at the compartment boundary and allowed to explore the box for 900 seconds. The time the rats spent in each compartment was recorded.

### Reinstatement procedure

At 9:30 on day 13, all the rats underwent an additional extinction trial. Rats of the MN group were randomly divided into 2 subgroups (RN and RS, n =10 each group). At 10:00, rats in both groups were given a priming injection of morphine (10 mg/kg, i.p.). Four hours later, each rat in the RN group was injected with naloxone (0.3 mg/kg, i.p.) and confined in its withdrawal-paired compartment for 30 minutes; rats in the RS group were given an equal volume of saline with the same procedure. At 10:00 on day 14, each rat was placed at the compartment boundary and allowed to explore the box for 900 seconds. The time the rats spent in each compartment was recorded.

### Statistical analyses

Naloxone-precipitated morphine withdrawal-induced CPA scores, defined as the time spent in the withdrawal-paired compartment during the test session minus the time spent in the same compartment on day 5 (preconditioning session), were the main outcome measures. The differences between the groups were tested using an analysis of variance (ANOVA) followed by Bonferroni’s post hoc test. Independent-samples t-tests were used to test differences between 2 specific groups.

Counts of naloxone-precipitated morphine withdrawal signs were regarded as continuous variables, and differences in these counts between the 3 groups were tested using ANOVA followed by Bonferroni’s post hoc test. The prevalence of diarrhea was presented as proportions of rats showing positive signs among all tested rats, and differences between these proportions were tested by Fisher’s exact probability test. A two-sided *P*<0.05 was regarded as statistically significant. SPSS 16.0 was used for all statistical analyses.
